# Protection against Oxidative Stress by Coenzyme Q10 in a Porcine Retinal Degeneration Model

**DOI:** 10.3390/jpm14040437

**Published:** 2024-04-22

**Authors:** Leonie Deppe, Ana M. Mueller-Buehl, Teresa Tsai, Carl Erb, H. Burkhard Dick, Stephanie C. Joachim

**Affiliations:** 1 Experimental Eye Research Institute, University Eye Hospital, Ruhr-University Bochum, In der Schornau 23-25, 44892 Bochum, Germany; leonie.deppe@rub.de (L.D.); ana.mueller-buehl@rub.de (A.M.M.-B.); teresa.tsai@rub.de (T.T.); burkhard.dick@kk-bochum.de (H.B.D.); 2 Private Institute for Applied Ophthalmology, Eye Clinic at Wittenbergplatz, 10787 Berlin, Germany; erb.glaukom@gmail.com

**Keywords:** glaucoma, coenzyme Q10, oxidative stress, neuroprotection, porcine organ culture

## Abstract

Oxidative stress plays an important role in neurodegenerative diseases, including glaucoma. Therefore, we analyzed if the antioxidant coenzyme Q10 (CoQ10), which is also commercially available, can prevent retinal degeneration induced by hydrogen peroxide (H_2_O_2_) in a porcine organ culture model. Retinal explants were cultivated for eight days, and H_2_O_2_ (500 µM, 3 h) induced the oxidative damage. CoQ10 therapy was applied (700 µM, 48 h). Retinal ganglion cells (RGCs) and microglia were examined immunohistologically in all groups (control, H_2_O_2_, H_2_O_2_ + CoQ10). Cellular, oxidative, and inflammatory genes were quantified via RT-qPCR. Strong RGC loss was observed with H_2_O_2_ (*p* ≤ 0.001). CoQ10 elicited RGC protection compared to the damaged group at a histological (*p* ≤ 0.001) and mRNA level. We detected more microglia cells with H_2_O_2_, but CoQ10 reduced this effect (*p* = 0.004). Cellular protection genes (*NRF2*) against oxidative stress were stimulated by CoQ10 (*p* ≤ 0.001). Furthermore, mitochondrial oxidative stress (*SOD2*) increased through H_2_O_2_ (*p* = 0.038), and CoQ10 reduced it to control level. Our novel results indicate neuroprotection via CoQ10 in porcine retina organ cultures. In particular, CoQ10 appears to protect RGCs by potentially inhibiting apoptosis-related pathways, activating intracellular protection and reducing mitochondrial stress.

## 1. Introduction

Until today, researchers have faced unanswered questions regarding degeneration in multifactorial retinal diseases like age-related macular degeneration, diabetic retinopathy, or glaucoma [1,2,3]. This has largely affected the progress of therapy and limited the current possibilities of curing these diseases.

Glaucoma represents the second most common cause for irreversible vision loss worldwide [1,4]. In Germany, about 1–2% of the population suffer from this disease. The number of patients worldwide will further rise in the next years, which will also drastically increase the cost of treatment [5,6]. Hence, there is an urgent need for new insights into glaucoma pathology. The progressive loss of retinal ganglion cells (RGCs) and degeneration of the optic nerve head result from multiple factors. Stressors considered to be involved in glaucoma development are oxidative stress, hypoxia, and inflammation [4,7,8,9,10]. One of the most common risk factors is high intraocular pressure (IOP) [4]. So far, several explanatory approaches of glaucoma pathology were made, but it is not yet fully understood [11,12].

Animal models are commonly used to elucidate disease pathologies, but the development of alternative methods, in adherence with the 3R principles (replace, reduce, and refine), is on the rise [13]. Ex vivo organ culture systems are one alternative. Here, explants can be cultivated for a restricted period of time [14]. Retinal explants can be gained from eyes as a byproduct of the food industry. For ophthalmic research, pig eyes offer several advantages. Besides their size and comparable morphology, their anatomy and cellular structure are comparable to those of humans. Pigs have a visual streak that is similar to the human macula [15]. Additionally, they possess two different opsins and are therefore able to detect color. These analogous structures are not found in nocturnal rodents [16].

Previously, we established a retinal organ culture system [17,18,19,20]. Here, hydrogen peroxide (H_2_O_2_) was used as an oxidizing agent [21]. Moreover, this organ culture model was used to examine protective agents like extremolytes, an iNOS-inhibitor, or hypothermia [18,20,22].

Now, we have investigated whether coenzyme Q10 (CoQ10), as a strong antioxidant, protects against retinal damage caused by H_2_O_2_. CoQ10 is involved in the mitochondrial energy supply and works as an electron carrier at mitochondrial multiprotein membrane complexes. It is indispensable for ATP synthesis [23,24]. In addition, CoQ10 is present in blood serum and cell membranes. Endogenous CoQ10 is synthesized via the mevalonate metabolic pathway and protects against free radicals. Normally, production decreases with advanced age, but it can also be upregulated under pathological conditions [25]. Furthermore, CoQ10 can influence gene expression, signal transduction, and metabolism, indicating a possibility of retinal protection in experimental glaucoma [26,27,28]. Interestingly, CoQ10 was identified as a successful therapeutic approach in other glaucoma animal models using ischemic conditions or intravitreal injections [29,30]. However, the exact mechanism of neuroprotection in glaucoma is still unclear and should be further analyzed. Hence, in the current study, we investigated for the first time if CoQ10 has neuroprotective potential in a retinal organ culture. To our knowledge, a project of this kind has not been carried out before and promises unique insights into the work mechanism of the antioxidant. This novel type of utilizing porcine tissue, which is quite similar to human tissue, is particularly helpful here. This will allow further steps to be taken towards the performance of clinical studies and applicable therapy, which are urgently needed in the field of glaucoma.

## 2. Materials and Methods

### 2.1. Preparation of Porcine Retinal Explants and Oxidative Stress Induction

Porcine eyes were obtained from the local slaughterhouse. Authorization from the veterinary office is available under the registration number DE05911002921. Tissue preparation was performed as described previously [17,20]. Briefly, eye cups were cut into quarters to obtain a shamrock-like structure. In three of the quarters, a round piece of retina was punched out and placed on an insert with RGCs facing up (Disposable Biopsy Punch, Ø 6 mm, KAI MEDICAL, Solingen, Germany). Each piece of tissue was used as an independent sample, placed in a different study group, and was treated according to the three different groups. All explants were cultured on filter inserts (30 mm diameter, Merck Millipore, Co Cork, Ireland) in 6-well plates (CELLSTAR^®^, Frickenhausen, Germany). A total of 1 mL of Neurobasal A medium supplemented with 0.8 mM L-glutamine, 2% B27, 1% N2 (all Gibco^®^ Thermo Fischer Scientific, Waltham, MA, USA), and 2% penicillin/streptomycin (Sigma-Aldrich Chemie GmbH, Steinheim, Germany) was added to each well. The explants were cultured for eight days at 37 °C and 5% CO_2_ (n = 14/group).

**Figure 1:**
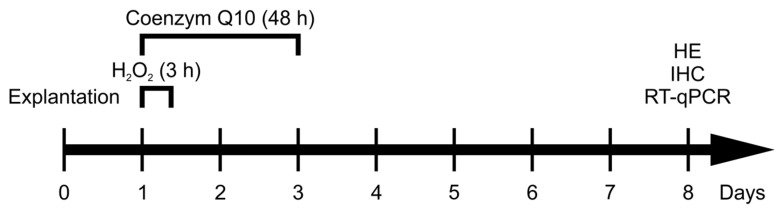
Experimental setup. Retinal explants were prepared on day zero. Degeneration was induced on day one by H_2_O_2_ (500 µM) for 3 h. The H_2_O_2_ + CoQ10 explants simultaneously received CoQ10 treatment (700 µM), which remained for 48 h. Retinae of the control group received medium without stressor or CoQ10. After eight days of cultivation, samples from all three groups were analyzed with hematoxylin and eosin staining (HE), immunohistological staining (IHC), and quantitative real-time PCR (RT-qPCR) analysis.

Degeneration in all damaged groups was induced by adding 500 µM H_2_O_2_ to the medium (Sigma Aldrich, St. Louis, MO, USA) at day one for three hours. The concentration was based on unpublished pilot studies and matching literature [17]. Simultaneously, 700 µM of CoQ10 (Sigma Aldrich, St. Louis, MO, USA), diluted in Lutrol (Sigma Aldrich, St. Louis, MO, USA), was added into the therapy group for 48 h (Figure 1). The choice of the CoQ10 concentration was also based on unpublished preliminary studies of our working group in this model. Further pilot tests showed that the use of Lutrol as a solvent for CoQ10 had no effect on the retinal explants. In general, all added substances could be dissolved in the medium described above. The control group received the same amount of medium without stressor or therapy. In total, the study consisted of three groups: control, H_2_O_2_, and H_2_O_2_ + CoQ10.

The medium was changed once a day on days zero to three and on the fifth and seventh day. For (immune-) histology (n = 8/group), the tissue was fixed with 4% PFA and dehydrated with 15 and 30% sucrose/PBS solution after eight days. The fixed tissue was embedded and stored at −80 °C. Samples for the RT-qPCR (n = 6/group) analyses were directly frozen at 80 °C.

### 2.2. (Immuno-) Histological Staining of Retinal Cross-Sections

Retinal explants (n = 8/group) were cut into 10 µm thick cross-sections with the Microtom HM 560 (Thermo Scientific, Darmstadt, Germany).

Three sections per explant were stained with hematoxylin + eosin (HE) [31]. The thickness of the retina was measured in three different areas of two photos per section via ZEN 2012 imaging software (Carl Zeiss MicroImaging GmbH, Oberkochen, Germany). Six images per explant were analyzed, and the mean value was used for statistics.

Immunofluorescence analyses of RGCs and microglia were performed using specific primary antibodies against the RNA-binding protein with multiple splicing (RBPMS) and the ionized calcium-binding adapter molecule 1 (Iba1) (Table 1) [32,33,34]. First, retinal cross-sections (6/explant) were dried at 37 °C for 10 min and rehydrated in 1xPBS for 4 min. The blocking solution consisted of 20% normal donkey serum, 1% bovine serum albumin, and 0.2% TritonX diluted in 1xPBS (1 h). Subsequently, the primary antibody was applied overnight at room temperature, and the secondary antibody was incubated the next day in darkness for 1 h at room temperature. Both antibodies were dissolved in blocking solution. 4′,6′-Diamidin-2-phenylindol (DAPI; 1:10) was applied for 5 min to visualize cell nuclei. Finally, slides were capped with Immu-Mount. Four images were taken of each cross-section with the fluorescence microscope (Axio Imager M2, Zeiss, Oberkochen, Germany) at 400× magnification. To evaluate the number of positive cells, 24 images of each explant were cropped to a defined size (800 × 600 pixel). Only specific signals co-localized with DAPI were counted (ImageJ 1.44 M; NIH, Bethesda, MD, USA). Iba1^+^ cells were counted in the ganglion cell layer, inner plexiform layer, and inner nuclear layer together as well as in the ganglion cell complex (GCC; ganglion cell layer and inner plexiform layer).

### 2.3. RNA Isolation and cDNA Synthesis

RNA isolation was performed according to the manufacturer’s instructions of the GeneEluteTM Mammalian Total RNA Miniprep Kit (Sigma-Aldrich, St. Louis, MO, USA). Therefore, retinal tissue (n = 6/group) was lysed with a mixture of 2-mercaptoethanol. Afterwards, the RNA concentration was measured using a NanoDropTM One spectrophotometer (Thermo Fisher Scientific, Madison, WI, USA). A total of 1 µg of RNA was used for cDNA synthesis, which was performed with the First Strand cDNA Synthesis Kit (Thermo Fisher Scientific, Vilnius, Lithuania).

### 2.4. Quantitative Real-Time PCR (RT-qPCR)

RT-qPCR evaluations based on the SYBR Green I protocol were carried out with the PikoRealTM 96 Real-Time Thermal Cycler (Thermo Fisher Scientific, Vantaa, Finland). Duplicates for every cDNA and primer pair were included. All used nucleotide sequences were researched on NCBI, and primers were blasted with the offered tool (Table 2). The final volume in each 96-well plate well amounted to 20 µL, consisting of 5 µL of cDNA and 15 µL of primer mix. Ct values were calculated via PikoReal 2.2. The results were normalized to the housekeeping gene *Histocompatibility 3* (*H3*). Both the difference between the control and H_2_O_2_ groups and among the H_2_O_2_ groups was examined.

### 2.5. Statistical Analyses

Regarding (immuno-) histological data, groups were compared by one-way ANOVA, followed by Turkey’s honest post hoc test (Statistica V13, StatSoft, Hamburg, Germany), and they are presented as mean ± SEM. The RT-qPCR results were analyzed with the 2^−ΔΔCT^ method and are shown as mean ± SEM, with all single samples presented as dots. Afterwards, the fold expression was also compared with the Statistica software (version 14.0.1.25). A *p* value less than 0.05 was considered statistically significant: * *p* ≤ 0.05, ** *p* ≤ 0.01, and *** *p* ≤ 0.001 compared to the controls and ^#^ *p* ≤ 0.05, ^##^ *p* ≤ 0.01, and ^###^ *p* ≤ 0.001 compared to H_2_O_2_.

## 3. Results

### 3.1. HE Staining Revealed No Significant Changes in Retinal Thickness

**Figure 2:**
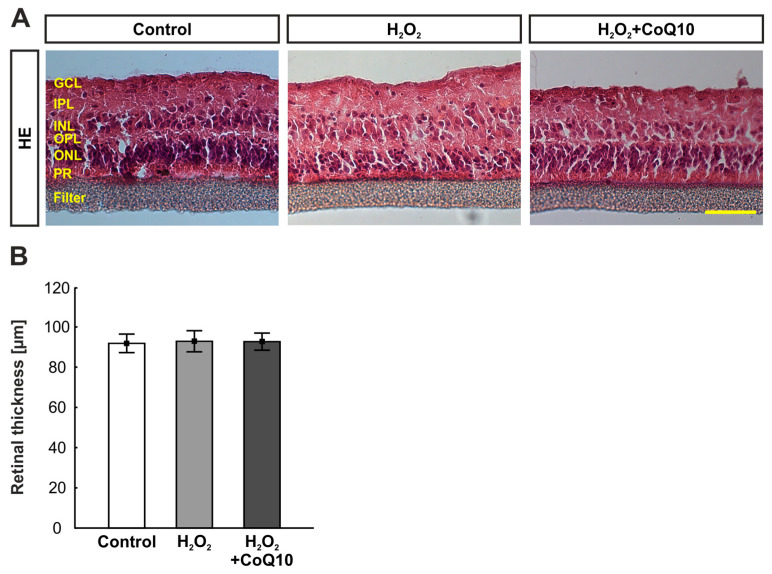
Retinal thickness was comparable in all groups. (**A**) Pictures displaying the retinal layers on HE-stained sections of each experimental group after 8 days. (**B**) No alteration in retinal thickness was noted within the groups. CoQ10 = coenzyme Q10; GCL = ganglion cell layer; HE = hematoxylin and eosin; INL = inner nuclear layer; IPL = inner plexiform layer; ONL = outer nuclear layer; OPL = outer plexiform layer; PR = photoreceptors. n = 8/group, values are shown as mean ± SEM. Scale bar: 50 µm.

To investigate the macrostructure of the cultured retina, layer thickness was measured after HE staining. The total retinal thickness (GCL to ONL) of the H_2_O_2_-damaged and CoQ10-treated groups was compared to the control group (Figure 2A). There were no statistical differences between the groups (control: 91.85 ± 4.50 µm; H_2_O_2_: 92.89 ± 5.25 µm, *p* = 0.999; H_2_O_2_ + CoQ10: 92.71 ± 4.26 µm, *p* = 0.999; Figure 2B).

### 3.2. CoQ10 Minimized RGC Loss

**Figure 3:**
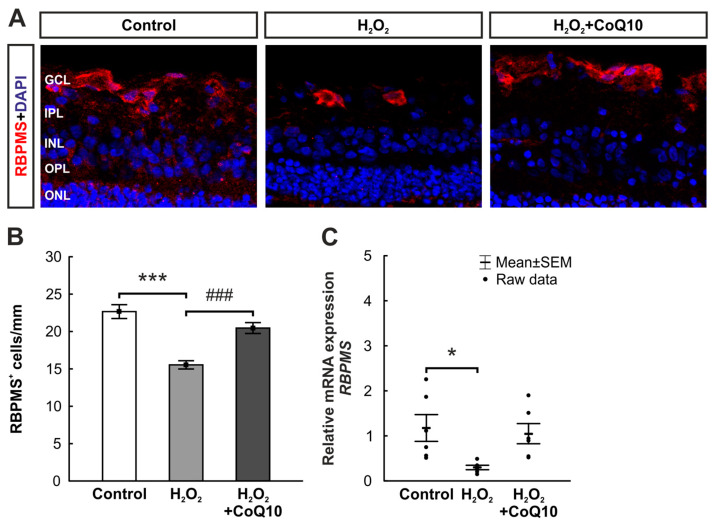
RGC protection through CoQ10 after H_2_O_2_ exposition. (**A**) RGCs of all groups were stained with antibodies against RBPMS (red) after eight days. DAPI (blue) was used to visualize cell nuclei. (**B**) RGC number was significantly decreased in the H_2_O_2_ group compared to controls (*p* ≤ 0.001). This RGC loss was no longer detectable after CoQ10 treatment compared to the damaged group (*p* ≤ 0.001). (**C**) The relative mRNA level of *RBPMS* was significantly decreased after oxidative stress induction compared to controls (*p* = 0.03), while the expression in the H_2_O_2_ + CoQ10 group was similar to the control group. CoQ10 = coenzyme Q10; GCL = ganglion cell layer; INL = inner nuclear layer; IPL = inner plexiform layer; ONL = outer nuclear layer; OPL = outer plexiform layer. (**B**): n = 8/group, values are shown as mean ± SEM; (**C**): n = 6/group, values are shown as mean ± SEM, raw data visualized as dots. Scale bar: 20 µm. * *p* ≤ 0.05 and *** *p* ≤ 0.001 vs. control and ^###^ *p* ≤ 0.001 vs. H_2_O_2_.

Sections of retinae were stained against RBPMS (Figure 3A). Significant RGC loss was identified in the H_2_O_2_ group (15.54 ± 0.55 cells/mm, *p* ≤ 0.001) compared to controls (22.66 ± 0.93 cells/mm), which was no longer detectable with the CoQ10 treatment (20.46 ± 0.73 cells/mm, *p* ≤ 0.001; Figure 3B). 

RT-qPCR analyses verified these results and presented a significantly decreased mRNA expression of *RBPMS* in the H_2_O_2_ group compared to the control group (0.3-fold expression ± 0.05, *p* = 0.03). *RBPMS* expression in the H_2_O_2_ + CoQ10 group was similar to controls (1.05-fold expression ± 0.22, *p* = 0.912). H_2_O_2_ + CoQ10 samples showed a non-significant increased *RBPMS* expression compared to H_2_O_2_ group (3.78-fold expression ± 0.8, *p* = 0.066; Figure 3C).

### 3.3. Reduced Microglia Accumulation by CoQ10 after Eight Days

**Figure 4:**
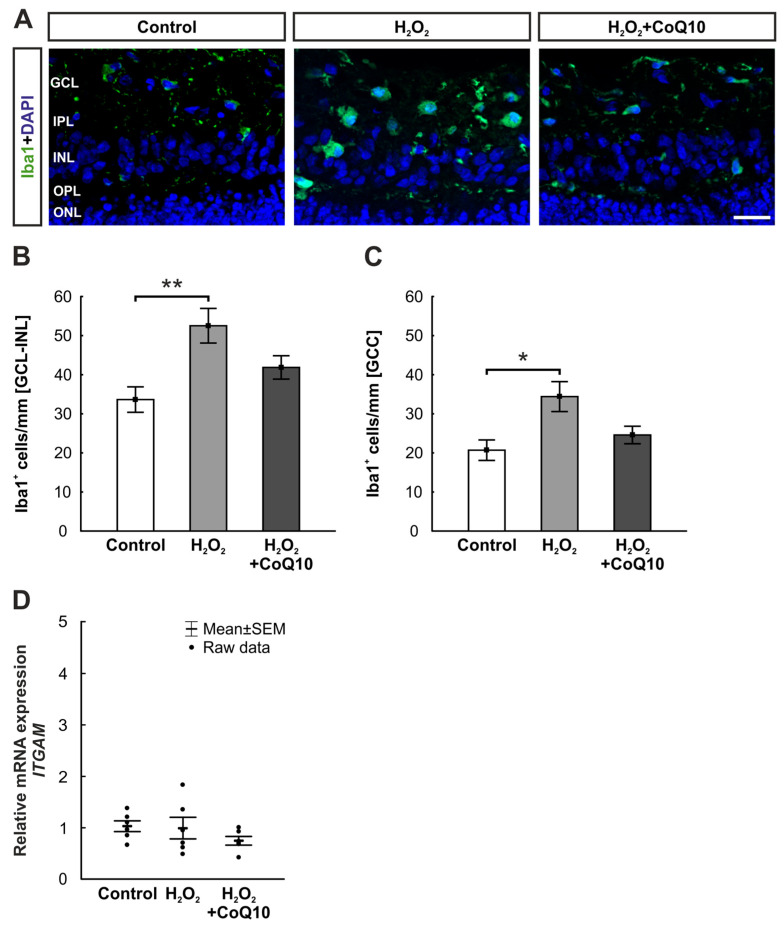
CoQ10 prevents H_2_O_2_-induced proliferation of microglia. (**A**) After eight days, microglia cells were stained with antibodies against Iba1 (green) and DAPI (blue), which was used to visualize the cell nuclei. (**B**) The H_2_O_2_ group revealed a significant increase in Iba1^+^ cells in the area of the GCL to the INL compared to the controls (*p* = 0.003). In the H_2_O_2_ + CoQ10 group, no changes were visible. (**C**) When comparing the GCC of the damaged group with the one of the controls, similar results were detectable (*p* = 0.010). (**D**) In terms of mRNA level, the expression of *ITGAM* revealed no changes in any groups. Also, the comparison between both H_2_O_2_ groups was unremarkable. CoQ10 = coenzyme Q10; GCC = ganglion cell complex; GCL = ganglion cell layer; INL = inner nuclear layer; IPL = inner plexiform layer; ONL = outer nuclear layer; OPL = outer plexiform layer. (**B**,**C**): n = 8/group, values are shown as mean ± SEM; (**D**): n = 6/group, values are shown as mean ± SEM, raw data visualized as dots. Scale bar: 20 µm. * *p* ≤ 0.05 and ** *p* ≤ 0.01 vs. control.

Microglia were also examined in the inner retina after eight days using an Iba1 antibody (Figure 4A). Cell counting in the ganglion cell layer (GCL) to the inner nuclear layer (INL) showed significantly more Iba1^+^ cells after oxidative stress induction (52.53 ± 4.42 cells/mm, *p* = 0.004) compared to controls (33.64 ± 3.25 cells/mm). This was not detectable after CoQ10 treatment (41.86 ± 2.99 cells/mm, *p* = 0.264; Figure 4B). The same alterations were observable when looking exclusively at the ganglion cell complex (GCC) consisting of GCL and inner plexiform layer (IPL) (control: 20.69 ± 2.62 cells/mm; H_2_O_2_: 34.4 ± 3.83 cells/mm, *p* = 0.010; H_2_O_2_ + CoQ10: 24.58 ± 2.24 cells/mm, *p* = 0.631; Figure 4C).

In contrast to the histological evaluation, no statistically relevant changes were found within the RT-qPCR analysis. The relative mRNA expression of *integrin alpha M* (*ITGAM*), a specific microglia gene which codes for the cell adhesion molecule integrin CD11b, was comparable between the groups (H_2_O_2_: 0.99-fold expression ± 0.21, *p* = 0.983; H_2_O_2_ + CoQ10: 0.74-fold expression ± 0.08, *p* = 0.370). Comparing both H_2_O_2_ groups also showed no differences (H_2_O_2_ + CoQ10: 0.84-fold expression ± 0.09, *p* = 0.465; Figure 4D).

### 3.4. Inflammatory Activity Was Not Affected by CoQ10

**Figure 5:**
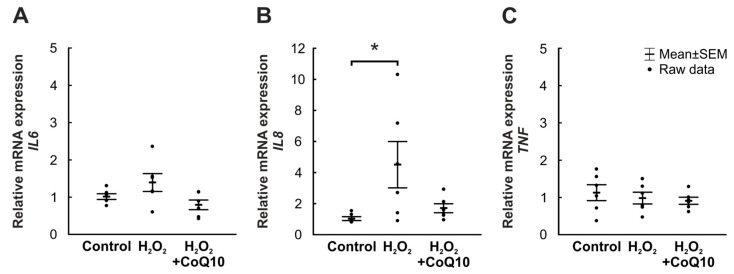
Low inflammatory response after eight days through H_2_O_2_ or CoQ10 treatment. (**A**) Both groups showed no significant changes in relative *IL6* mRNA expression versus controls. (**B**) In contrast, the relative mRNA expression of *IL8* was significantly increased in the H_2_O_2_ group compared to the control group (*p* = 0.035), but the H_2_O_2_ + CoQ10 group showed no differences. Also, there were no differences in *IL8* expression in the direct comparison of the two H_2_O_2_ groups. (**C**) The relative mRNA expression of *TNF* was not altered. CoQ10 = coenzyme Q10. n = 6/group, values are shown as mean ± SEM, raw data visualized as dots. * *p* ≤ 0.05 vs. control.

To investigate the inflammatory response, the relative mRNA expression of specific cytokines was analyzed. In the case of *interleukin 6* (*IL6*), expression levels were similar in all groups (H_2_O_2_: 1.39-fold expression ± 0.24, *p* = 0.262; H_2_O_2_ + CoQ10: 0.79-fold expression ± 0.13, *p* = 0.612). The comparison of both H_2_O_2_ groups also revealed no significant differences (H_2_O_2_ + CoQ10: 0.62-fold expression ± 0.1, *p* = 0.051; Figure 5A). 

Elevated expression patterns were found for *interleukin 8* (*IL8*) compared to controls. The *IL8* expression level of the H_2_O_2_ group (4.51-fold expression ± 1.49, *p* = 0.035) was significantly elevated, while the H_2_O_2_ + CoQ10 (1.7-fold expression ± 0.29, *p* = 0.855) displayed no increased expression. Accordingly, the H_2_O_2_ + CoQ10 expression was similar to the control group. The comparison of both damaged groups did not reveal any differences (0.53-fold expression ± 0.09, *p* = 0.095; Figure 5B).

No changes could be detected regarding *tumor necrosis factor* (*TNF*) expression levels (H_2_O_2_: 0.98-fold expression ± 0.16, *p* = 0.806; H_2_O_2_ + CoQ10: 0.91-fold expression ± 0.09, *p* = 0.623). The comparison of the two H_2_O_2_ groups also did not expose any alterations (1.00-fold expression ± 0.10, *p* = 0.948; Figure 5C).

### 3.5. CoQ10 Stimulates Intracellular Protection Cascades against Oxidative Stress

**Figure 6:**
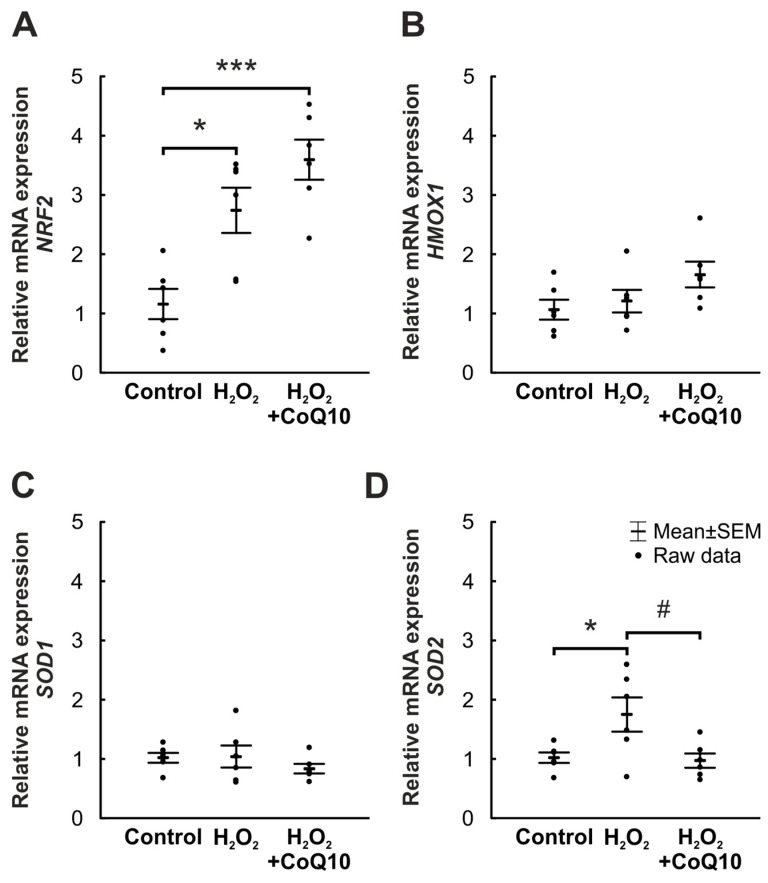
CoQ10 stimulates intracellular protection cascade against oxidative stress. (**A**) Relative expression of *NRF2* was significantly increased after H_2_O_2_ and CoQ10 in comparison to controls (H_2_O_2_: *p* = 0.011; H_2_O_2_ + CoQ10: *p* ≤ 0.001). In a direct comparison of both H_2_O_2_ groups, the *NRF2* expression was not significantly changed. (**B**) Comparable but not significant results were detectable considering the *HMOX1* expression, where both groups exposed a higher expression level compared to the controls. However, the variance with CoQ10 was not strong enough to cause differences while comparing the two H_2_O_2_ groups. (**C**) No statistical differences were noted regarding the *SOD1* expression when comparing the H_2_O_2_ groups to the control group. *SOD1* expression in the two H_2_O_2_ groups was similar. (**D**) In the H_2_O_2_ group, the expression of *SOD2* was significantly increased (*p* = 0.038). The H_2_O_2_ + CoQ10 group showed a significantly decreased *SOD2* expression compared to the H_2_O_2_ group (*p* = 0.026). CoQ10 = coenzyme Q10. n = 6/group, values are shown as mean ± SEM, raw data visualized as dots. * *p* ≤ 0.05 and *** *p* ≤ 0.001 vs. control; # *p* ≤ 0.05 vs. H_2_O_2_.

Different gene expression patterns regarding oxidative stress and intracellular protection cascades were analyzed by RT-qPCR. The *nuclear factor erythroid 2 (NF-E2)-related factor 2* (*NRF2*) mRNA level, which initiates protection against oxidative stress, increased significantly after oxidative stress and was more prominent with CoQ10 treatment compared to controls (H_2_O_2_: 2.74-fold expression ± 0.38, *p* = 0.011; H_2_O_2_ + CoQ10: 3.6-fold expression ± 0.34, *p* ≤ 0.001). But the H_2_O_2_ + CoQ10 group showed no significant *NRF2* elevation compared to the H_2_O_2_ group (1.39-fold expression ± 0.13, *p* = 0.192; Figure 6A).

Similar results, which were not significant, could be found regarding *heme oxygenase 1* (*HMOX1*), one of the enzymes activated by Nrf2. The H_2_O_2_ group displayed an *HMOX1* expression similar to controls (1.21-fold expression ± 0.19, *p* = 0.861), while the H_2_O_2_ + CoQ10 group had a slightly, but not significant, increased expression (1.66-fold expression ± 0.22, *p* = 0.110). The comparison of the two H_2_O_2_ groups showed no difference (H_2_O_2_ + CoQ10: 1.45-fold expression ± 0.19, *p* = 0.257; Figure 6B).

*Superoxide dismutase 1* (*SOD1*) is an important enzyme for cellular protection against oxidative stress and is predominantly localized in the cytoplasm [35,36,37]. *SOD1* gene expression showed no significant changes in all groups (H_2_O_2_: 1.04-fold expression ± 0.19, *p* = 0.992; H_2_O_2_ + CoQ10: 0.84-fold expression ± 0.08, *p* = 0.575). The comparison of both H_2_O_2_ groups could not reveal any variations (H_2_O_2_ + CoQ10: 0.87-fold expression ± 0.08, *p* = 0.503; Figure 6C).

Another type of protective superoxide dismutase, which occurs in the mitochondrial matrix, is *superoxide dismutase 2* (*SOD2*) [35,36,37]. A significantly increased mRNA expression of *SOD2* was identified in the H_2_O_2_ group (1.75-fold expression ± 0.29, *p* = 0.038) compared to controls, which was not detectable after CoQ10 application (0.97-fold expression ± 0.12, *p* = 0.981). Comparing the two damaged groups, CoQ10 was able to reduce the *SOD2* expression significantly (0.6-fold expression ± 0.08, *p* = 0.026; Figure 6D).

## 4. Discussion

To date, glaucoma patients have mainly been treated through IOP lowering, while neuroprotective agents still need to be identified. Reliable models are needed to improve our knowledge of multifactorial glaucoma pathogenesis [38,39]. Therefore, we used a specific ex vivo organ culture system of porcine eyes, where neurodegeneration can be stimulated via oxidative stress induced with H_2_O_2_ [17,20,40]. We investigated if it is possible to counteract this damage with the antioxidant CoQ10 [23,24,26,27,28]. Our presented results demonstrate a positive effect of CoQ10 against oxidative stress in a porcine ex vivo organ culture model for the first time. We could identify strong RGC protection, an inhibition of microglia accumulation, and a specific modulation of oxidative-stress-related genes. Due to the high similarity between humans and pigs, these new findings point to promising future projects with CoQ10 in the clinical field.

RGC analysis showed a protective effect of CoQ10. The reduced number of cells and the level of relative *RBPMS* mRNA expression after oxidative damage could be recovered with CoQ10. These results support our hypothesis that CoQ10 plays a neuroprotective role. The reason for the explicit RGC damage after H_2_O_2_ application may be related to its high energy turnover. Since RGCs have a particularly high energy consumption, they are sensitive to mitochondrial undersupply [41,42]. The breakdown of ATP synthesis leads to impaired function of RGCs, which may occur after mitochondria permeabilization. Moreover, pathological accumulation of ROS causes the release of mitochondrial dissolution factors, so that apoptosis and RGC death, which have been demonstrated here, are induced [43,44]. The fact that CoQ10 can eliminate oxidative stress, ROS, and maintain the mitochondrial membrane potential may account for the protective effect [25]. Thus, the specific RGC protection by CoQ10 is likely due to its close correlation to neuronal energy turnover and functionality in mitochondria. The pathological pathways during glaucoma usually lead to cell death and particularly affect RGCs [1,9]. This cell death can be triggered by apoptotic signaling cascades, which are either part of the physiological cell process or can lead to the activation of cellular self-destruction [43,44]. Neurodegenerative diseases are often characterized by an inappropriate rate of apoptosis after oxidative stress [19,44,45]. CoQ10 supplementation in mice was able to protect the quality of postovulatory aged oocytes [46]. It provided reversion of disorders of spindle assembly, chromosome misdirection, and abnormal distribution patterns in mitochondria. Consistent with our results, the addition of CoQ10 could reduce age-related oxidative stress and apoptosis [46]. Another study investigating CoQ10 protection in a murine Parkinson’s disease model with chronic exposure to rotenone also revealed reduced apoptosis and restoration of mitochondrial membrane potential [47]. This study again supports the changes found here in the glaucoma model and demonstrates the protective effects of CoQ10.

The Iba1 staining of the inner retina in our study demonstrated increased microglial presence in the H_2_O_2_ group. Microglia are part of the neuronal immune system with their phagocytotic work and have a protective function to some extent. Macrophages are highly agile, secrete cytokines, and migrate rapidly into damaged tissue [48,49]. Both the absence of microglial activity and the excessive presence of cells can lead to cell death [50,51,52]. The increased microglia number after H_2_O_2_ injury suggests an inflammatory response [17,53]. Similar studies with porcine retinae could also detect a late response of microglia after H_2_O_2_ damage, which was minimized through hypothermic conditions [20]. The anti-inflammatory effect of CoQ10 occurring in this study results from its antioxidant capabilities of the electron transfer and its redox capacity [23,24,25]. By scavenging oxygen free radicals, oxidative stress does not occur. No proinflammatory cytokines are produced, and the necessary signaling cascades for microglial activation are absent [54]. In this way, CoQ10 protects against microglial accumulation, inflammatory signaling, and cell death [55]. The lack of changes in the microglial mRNA expression could be due to the CoQ10 concentrations used, the time points, or a difference between cell presence and gene expression. Discrepancies between expression and cellular occurrence were already seen in other studies and might be related to post-transcriptional and translational regulations [31,56,57].

Various inflammatory markers were investigated since oxidative stress activates immunological processes [17,45,58,59]. Relative mRNA expression of *IL6*, *IL8*, and *TNF* genes were examined to analyze potential anti-inflammatory aspects of CoQ10. *IL8* expression was increased in H_2_O_2_ samples, whereas CoQ10 had a protective effect and could reduce the elevated expression to the control level. Previous studies using this porcine model showed an *IL8* increase after four days, indicating increased inflammatory activity after oxidative stress [45]. IL8 is secreted by microglial cells and can therefore be increased due to a stronger presence of these cells, as we have detected in our study [60,61]. A clinical study regarding CoQ10 as a treatment for acute viral myocarditis showed promising results. CoQ10 treatment appeared to have an anti-inflammatory effect, including a decrease in IL8 levels in the serum [62]. Furthermore, there are other sources of IL8 in the retina which were not considered here, like astrocytes and Müller cells [63,64,65]. It would be beneficial to use other concentrations, exposure times, or investigation time points in the future.

CoQ10 could trigger RGC protection via different pathways. Nrf2 as a transcription factor regulates the expression of oxygenase’s as Hmox1, protects against cellular stress, and induces the expression of other antioxidative genes [66,67]. Based on this functional linkage of the two, the similar expression patterns found here can be explained and a certain dependence can be justified even if the *HMOX1* expression changes were not significant. The fact that H_2_O_2_ alone also stimulated *NRF2* expression can probably be explained by a general reaction to oxidative stress, which was amplified to a high extent by CoQ10 [68]. A previous murine study regarding RGC loss demonstrated that pharmacological activation of Nrf2 has a positive effect on cell survival [69]. In this regard, activation of Nrf2 and Hmox1, which were positively affected by CoQ10 in this study, appears to have minimized RGC loss.

SODs are endogenous enzymes and play a pivotal role in preventing oxidative stress and ROS. They are key elements in the metabolization of oxygen [35]. Their expression can be altered by certain intracellular and extracellular triggers, like interleukin increase [37]. The elevated *IL8* expression due to oxidative stress could have been such a trigger, which in turn stimulated *SOD* expression via signaling pathways, as mentioned before [45,60,61,70]. SOD2 works predominantly in the mitochondrial matrix, whereas SOD1 is found in the cytoplasm [35,36]. Since oxidative stress in neurodegenerative diseases particularly affects mitochondrial function and ATP supply, protective mechanisms in mitochondria are specifically targeted [71]. It has been shown that CoQ10 can influence gene expression and signaling pathways, which could explain the direct influence on expression patterns [26,27,28]. By maintaining mitochondrial membrane potential, CoQ10 was able to minimize oxidative damage and thus restore the expression of *SOD2* [72].

It should be mentioned that the study presented here also has some limitations. The evaluation of the protein level was carried out using immunohistology to evaluate the cell number. To quantify these regulations, Western blot analysis should be carried out in follow-up studies to gain more information about the amount of protein in retinal explants.

## 5. Conclusions

**Figure 7:**
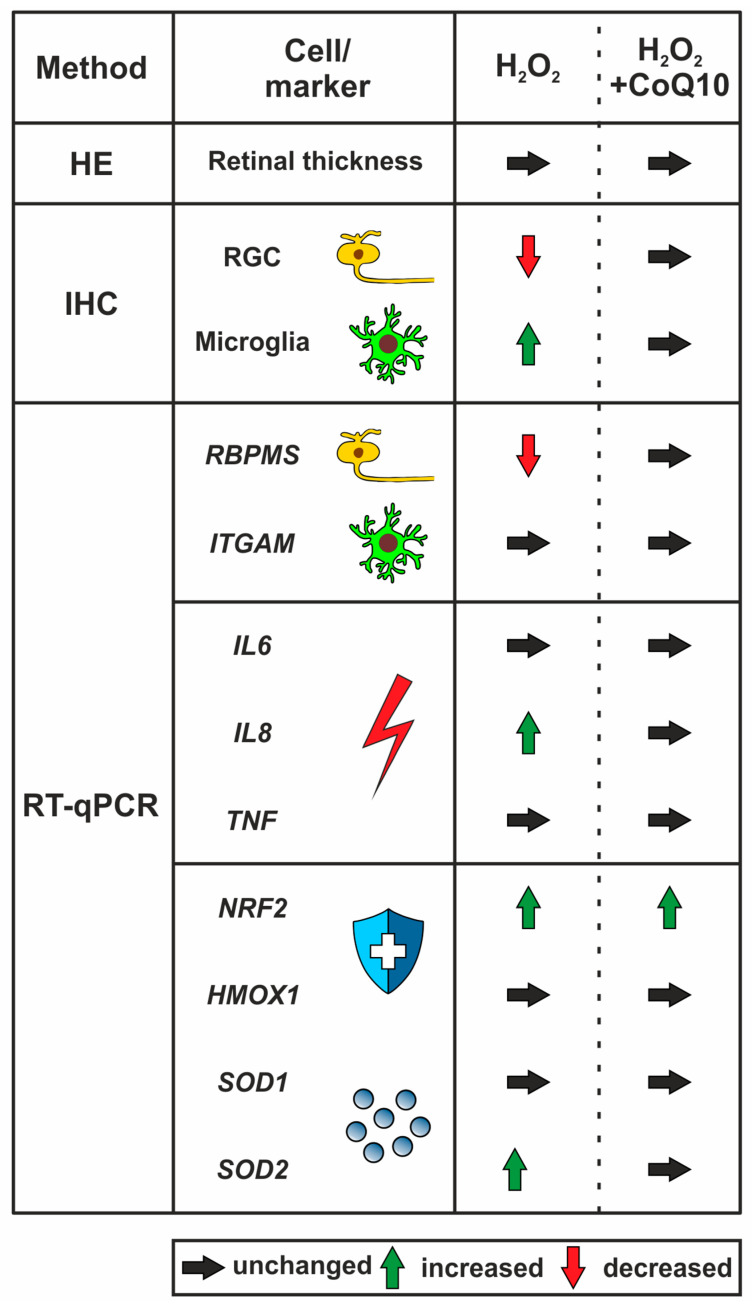
Graphical summary showing protective effects of CoQ10 on porcine retinae damaged by H_2_O_2_. Oxidative damage was induced by H_2_O_2_, and some samples were treated with CoQ10. A significant RGC loss through H_2_O_2_ application was noted via RBPMS in the immunohistological evaluation and RT-qPCR analysis, which was no longer detectable with CoQ10 treatment. Additionally, the damage caused higher microglia numbers identified by Iba1 staining. Within the analyses of inflammation markers, higher *IL8* mRNA levels were detected in the H_2_O_2_ group counteracted by CoQ10. Furthermore, the antioxidant was able to increase the expression of specific oxidative protection cascade genes, like *NRF2*, to a large extent. Finally, mitochondrial stress, examined by the expression of *SOD2*, was elevated after oxidative stress induction. This effect was no longer detectable after CoQ10 application. Overall, CoQ10 positively affected the protection of RGCs by possibly altering apoptotic-related processes, microglial activity, and oxidative stress, as well as associated protective cascades.

The aim of this work was to test the potential neuroprotective effect of CoQ10 in a porcine retinal organ culture. CoQ10 significantly prevented RGC death by likely decreasing apoptosis-related processes, reducing microglia proliferation, activating neuroprotective enzymes, and decreasing mitochondrial stress levels (Figure 7). Due to the association of the enzyme and mitochondrial activity, we hypothesize that the effective protection by CoQ10 arises from its influence on mitochondrial stability. Furthermore, this study illustrates the strong neuroprotective effect of CoQ10, specifically on RGCs. Overall, CoQ10 is a promising therapeutic approach for the treatment of glaucoma, and our work provides new information on its mechanisms of action.

## Figures and Tables

**Table 1:** Primary and secondary antibodies used for immunohistology.

Primary Antibodies				Secondary Antibodies		
Antibody	Source	Company	Dilution	Antibody	Company	Dilution
Anti-Iba1	Chicken	Synaptic Systems	1:500	Donkey anti-chicken Alexa Fluor 488	Jackson Immuno Research	1:500
Anti-RBPMS	Rabbit	Millipore	1:200	Donkey anti-rabbit Alexa Fluor 555	Invitrogen	1:500

**Table 2:** Sequences of primers used for RT-qPCR.

Gene	Primer Fwd (5′-3′) Primer Rev (5′-3′)	GenBank Acc. No.	Amplicon Size
*H3*	ACTGGCTACAAAAGCCGCTC	NM_213930.1	232
	ACTTGCCTCCTGCAAAGCAC		
*HMOX1*	GGCTGAGAATGCCGAGTTCA	NM_001004027.1	88
	GTGGTACAAGGACGCCATCA		
*IL6*	GCAGTCACAGAACGAGTGGA	NM_214399.1	84
	CTCAGGCTGAACTGCAGGAA		
*IL8*	TTCCAAACTGGCTGTTGCCT	M86923.1	178
	ACAGTGGGGTCCACTCTCAA		
*ITGAM*	AGAAGGAGACACCCAGAGCA	XM_021086380.1	169
	GTAGGACAATGGGCGTCACT		
*NRF2*	GCCGACTATTCCCAGGTAGC	XM_003133500.6	713
	GTTGTGCTTTCACGGTGGTC		
*RBPMS*	CGAGAAGGAGAACACCCCGAAC	XM_003133393.4	549
	CAAAAGACAGGTGTGTTGGGC		
*SOD1*	AAAACATGGTGGGCCAAAGG	NM_001190422.1	72
	CCATCTTTGCCAGCAGTCAC		
*SOD2*	CAGCTCGAGCAGGAATCTGG	NM_214127.2	87
	CCATAGTCGTACGGCAGGTC		
*TNF*	GCCCTTCCACCAACGTTTTC	NM_214022.1	97
	CAAGGGCTCTTGATGGCAGA		

## Data Availability

The datasets generated and/or analyzed during the current study are available from the corresponding author on reasonable request.
